# Copper-doped lanthanum manganite La_0.65_Ce_0.05_Sr_0.3_Mn_1−*x*_Cu_*x*_O_3_ influence on structural, magnetic and magnetocaloric effects

**DOI:** 10.1039/c7ra13244a

**Published:** 2018-02-13

**Authors:** M. Chebaane, R. Bellouz, Ma. Oumezzine, E. K. Hlil, A. Fouzri

**Affiliations:** Laboratory of Physical Chemistry of Materials, Faculty of Sciences of Monastir, University of Monastir 5019 Monastir Tunisia bellouzridha@yahoo.fr Oumezzine@hotmail.co.uk; Institut Néel, CNRS–Université J. Fourier B.P. 166 38042 Grenoble France

## Abstract

Bulk nanocrystalline samples of La_0.65_Ce_0.05_Sr_0.3_Mn_1−*x*_Cu_*x*_O_3_ (0 ≤ *x* ≤ 0.15) manganites are prepared by the sol–gel based Pechini method. The effect of the substitution for Mn with Cu upon the structural and magnetic properties has been investigated by means of X-ray diffraction (XRD), Raman spectroscopy and dc magnetization measurements. The structural parameters obtained using Rietveld refinement of XRD data showed perovskite structures with rhombohedral (*R*3̄*c*) symmetry without any detectable impurity phase. Raman spectra at room temperature reveal a gradual change in phonon modes with increasing copper concentration. The analysis of the crystallographic data suggested a strong correlation between structure and magnetism, for instance a relationship between a distortion of the MnO_6_ octahedron and the reduction in the Curie temperature, *T*_c_. A paramagnetic to ferromagnetic phase transition at *T*_C_ is observed. The experimental results confirm that Mn-site substitution with Cu destroys the Mn^3+^–O^2−^–Mn^4+^ bridges and weakens the double exchange (DE) interaction between Mn^3+^ and Mn^4+^ ions, which shows an obvious suppression of the FM interaction in the La_0.65_Ce_0.05_Sr_0.3_Mn_1−*x*_Cu_*x*_O_3_ matrix. The maximum magnetic entropy change −Δ*S*^max^_M_ is found to decrease with increasing Cu content from 4.43 J kg^−1^ K^−1^ for *x* = 0 to 3.03 J kg^−1^ K^−1^ for *x* = 0.15 upon a 5 T applied field change.

## Abstract 2

Bulk nanocrystalline samples of La_0.65_Ce_0.05_Sr_0.3_Mn_1−*x*_Cu_*x*_O_3_ (0 ≤ *x* ≤ 0.15) manganites are prepared by the sol–gel based Pechini method.

## Graphical abstract

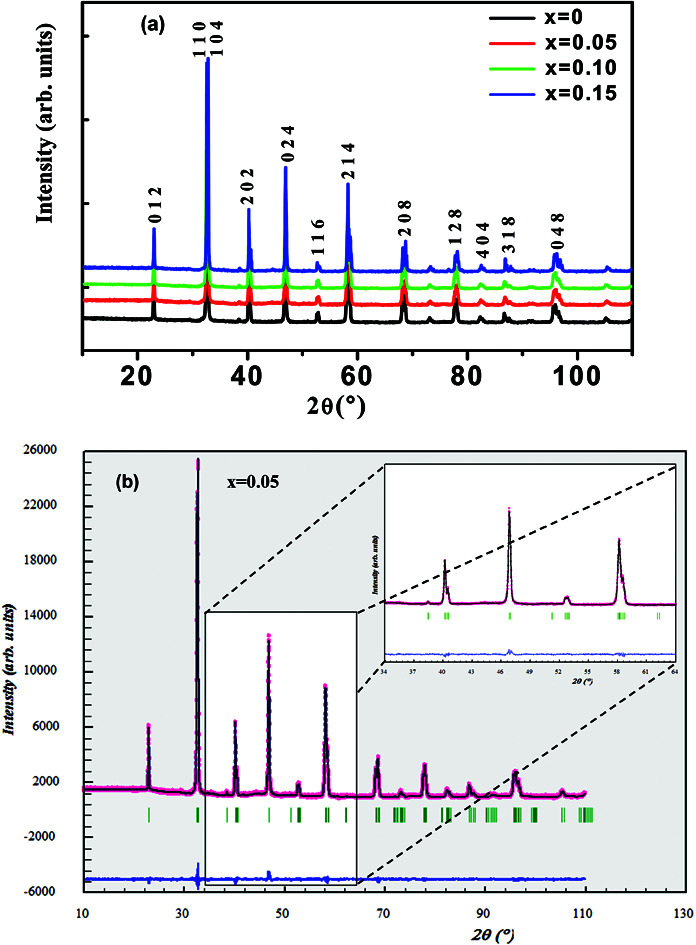

## 1. Introduction

Recently, perovskite manganites of R_1−*x*_A_*x*_MnO_3_ (where R and A are trivalent rare earth and divalent alkaline earth ions, respectively) have been the subject of intense research due to their interesting physical properties around the ferromagnetic (FM)–paramagnetic (PM) transition temperature (the Curie temperature, *T*_C_), such as the colossal magnetoresistance (CMR), the magnetocaloric effects (MCE) (related to a large magnetic entropy change) and the strong correlation between structural and magnetic properties. Doped lanthanum based manganites have been used in many technological applications, including magnetic recording, high-density data storage, hard disks, magnetic sensors, spin-electronic devices, and magnetic refrigerants.^1–4^

These materials offer a high degree of chemical flexibility leading to complex interplay between structural, electrical and magnetic properties. The double exchange (DE) effect in which e_g_ electrons transfer between adjacent Mn^3+^ and Mn^4+^ ions and the Jahn–Teller effect were used to understand FM–PM transition and CMR in manganites.^5,6^ A prominent feature of most manganites is that they will undergo a ferromagnetic–paramagnetic (FM–PM) phase transition at the Curie temperature *T*_C_ associated with an metal–insulator (M–I) transition at temperature *T*_MI_, which explains the fact that there exists a close relationship between the electrical and magnetic properties of manganites.^7,8^ Average ionic radius, electronic configuration, valance state and the concentration of the doping element are important parameters for tuning the magnetic and electronic properties of these materials.^9^

On the other hand, the synthesis technique greatly influences the physical and chemical characteristics of the rare-earth perovskite materials. There are various methods to synthesize the manganites compounds among them the Pechini sol–gel method. This method has been used successfully to produce high-quality specimens due to these potential advantages such as better homogeneities, lower processing temperatures, short annealing times, high purity of materials and improved material properties.

In manganites, it is possible to dope at both R-site and Mn-site, much research has been done on the substitution at the R-site with transition elements^10–12^ and/or rare-earths Eu,^13^ Ce,^14^ Pr,^15^ which can modify the Mn^3+^–O^2−^–Mn^4+^ network and in turn will intensively affect the intrinsic physical properties, such as ferromagnetism and (MCE). The substitution at the Mn site in perovskite oxides, with other transition metal ions,^16–20^ is more important because it not only modifies the Mn^3+^–O^2−^–Mn^4+^ network but also brings about many new exchange interactions between the Mn ion and the doped transition metal ions. Amongst the doping at Mn sites with transition elements, Cu substitution has been particularly investigated because of the special nature of its variable valence.^21–31^ In particular, Kim *et al.*^30,31^ reported the coexistence of Cu^2+^ and Cu^3+^ ions with Cu^2+^ dominant was found in their samples, and was thought to be responsible for the variations of the lattice parameters, Mn/Cu–O bond length and Mn/Cu–O–Mn/Cu bond angle. The doping of Cu changes the Cu^2+^/Cu^3+^ ratio and therefore the Mn^3+^/Mn^4+^ ratio, which may affect the electron carrier density, causing an intrinsic effect on the Mn^3+^–O^2−^–Mn^4+^ DE network.

The objective of this work was to synthesize nanocrystalline samples of La_0.65_Ce_0.05_Sr_0.3_Mn_1−*x*_Cu_*x*_O_3_ with an extended doping levels up to *x* = 0.15 and study the influence of copper-doping at Mn-site on the structural, magnetic and magnetocaloric properties.

## 2. Experimental procedure

### 2.1. Synthesis

Nanocrystalline samples of La_0.65_Ce_0.05_Sr_0.3_Mn_1−*x*_Cu_*x*_O_3_ (0 ≤ *x* ≤ 0.15) were prepared using the Pechini sol–gel method and a mixture of oxides and precursors, La(NO_3_)_3_·6H_2_O, Sr(NO_3_)_2_, Ce(NO_3_)_3_·6H_2_O, Mn_2_O_3_ and Cu(NO_3_)_2_·3H_2_O. The stoichiometric amounts of precursors were dissolved in distilled water at 90 °C and then a suitable amount of citric acid and ethylene glycol as coordinate agents were added. The resulting gel was pre-calcined (673 K for 3 h) to eliminate the organic material, ground and calcined again (973 K for 15 h) to eliminate the residual organic material. The obtained powder was then pressed into pellets (13 mm in diameter and 2–3 mm thick under a pressure of 5 ton cm^−2^). After that, the powder was sintered at 1173 K for 12 h in air.

### 2.2. Characterization

The morphological properties of the samples were investigated by scanning electron microscopy (SEM) on a JSM-6400 apparatus working at 20 kV. The structure and phase purity were checked by powder X-ray diffraction (XRD) using a "Panalytical X pert Pro" diffractometer with Cu K_α_ radiation (*k* = 1.5406 Å). Data for Rietveld refinement were collected in the range of 2 h from 10° to 120° with a step size of 0.017° and a counting time of 18 s per step. Raman scattering data was collected in the frequency range 100–1000 cm^−1^ using a Raman spectrometer. Magnetic measurements *versus* temperature and magnetic applied field were realized using a SQUID (Quantum Design) developed at Louis Neel Laboratory of Grenoble. The isothermals *M versus H* at various temperatures around *T*_C_ have been measured in applied fields up to 5 T.

## 3. Results and discussion

### 3.1. Structural properties

**Fig. 1:**
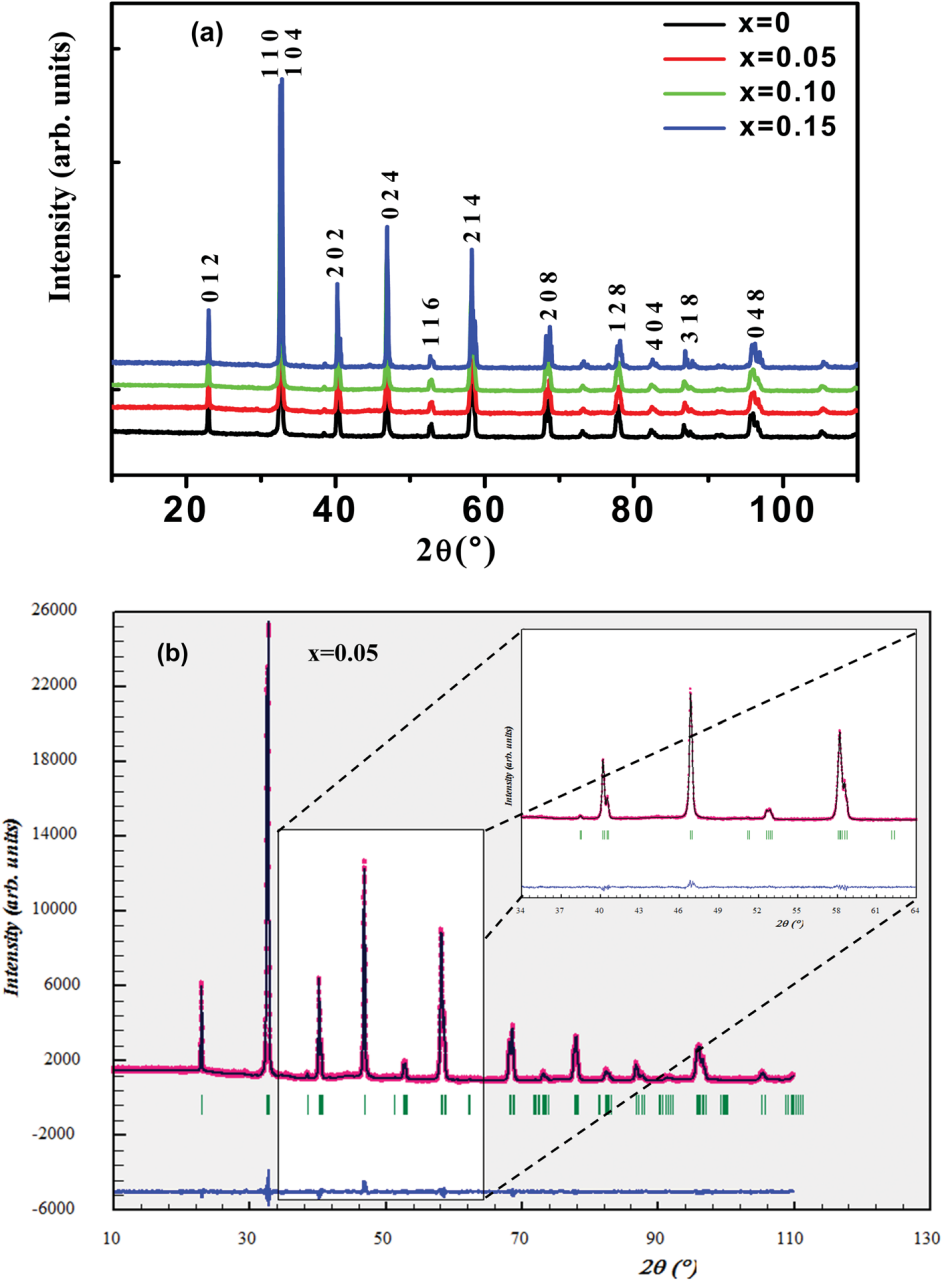
(a) XRD patterns of La_0.65_Ce_0.05_Sr_0.3_Mn_1−*x*_Cu_*x*_O_3_ (0 ≤ *x* ≤ 0.15) compounds at room temperature. (b) Rietveld refinement profile for *x* = 0.05 performed using FULLPROF. Open circles correspond to experimental data and the lines are fits. Vertical bars represent the Bragg reflections for the space group *R*3̄*c*. The difference pattern between the observed data and fits is shown at the bottom. The inset shows a zoom in the region between 2*θ*: 34–64°.

Fig. 1(a) shows the experimental XRD plots for the samples La_0.65_Ce_0.05_Sr_0.3_Mn_1−*x*_Cu_*x*_O_3_ with *x* = 0, 0.05, 0.1 and 0.15. We first discuss the structural parameters of the studied samples in present work. Using the Rietveld refinement method, we noted that all samples are single-phase with a rhombohedral structure of the *R*3̄*c* space group (no. 167), in which the (La, Ce, Sr) atoms are at 6a (0, 0, 1/4) positions, (Mn, Cu) at 6b (0, 0, 0) and O at 18e (*x*, 0, 1/4). These results are consistent with the values of the Goldschmidt tolerance factor *t*_G_:1
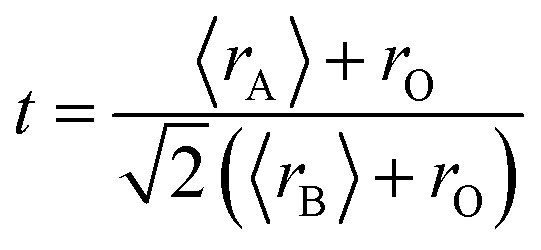
where *r*_A_, *r*_B_ and *r*_O_ are respectively the average ionic radii of A and B perovskite sites and of the oxygen anions. The tolerance factor is an important structural parameter, which reflects the local microscopic distortion from the ideal perovskite (ABO_3_) structure (*t* = 1), for which the B–O–B bond angle *θ* is equal to 180°. The values of *t*_G_ were estimated and listed in Table 1.

**Table 1:** Detailed results of Rietveld refinement of La_0.65_Ce_0.05_Sr_0.3_Mn_1−*x*_Cu_*x*_O_3_ (0 ≤ *x* ≤ 0.15) samples at room temperature

*x*	0	0.05	0.10	0.15
Space group	*R*3̄*c*	*R*3̄*c*	*R*3̄*c*	*R*3̄*c*
Lattice parameters				
*a* = *b* (Å)	5.5037 (1)	5.4995 (2)	5.4979 (2)	5.4954 (1)
*c* (Å)	13.3691 (2)	13.3549 (3)	13.3452(1)	13.3332 (3)
*V* (Å^3^)	350.70 (2)	349.79 (2)	349.29 (2)	348.71 (1)
(La, Ce, Sr) *B*_iso_ (Å^2^)	0.78 (3)	0.64 (3)	0.62 (3)	0.35 (4)
(Mn, Cu) *B*_iso_ (Å^2^)	0.29 (4)	0.18 (2)	0.14 (4)	0.13 (7)
(O) *B*_iso_ (Å^2^)	0.8 (1)	0.65 (1)	0.61 (1)	0.32(1)
(O) *x*	0.462 (1)	0.462 (4)	0.461 (2)	0.459 (1)
*d*_(Mn, Cu–O)_ (Å)	1.9518 (4)	1.9522 (5)	1.9533 (8)	1.9539 (8)
*θ*_(Mn, Cu–O–Mn, Cu)_ (°)	167.64 (2)	167.53 (2)	167.37 (3)	167.11 (5)
*W* (× 10^−2^) (u.a.)	9.570	9.562	9.554	9.552
*t*_G_	0.976	0.979	0.981	0.984
*T* ^DRX^_G_ (nm)	77	83	85	94
*R*_wp_ (%)	3.68	3.34	3.32	3.61
*R*_p_ (%)	2.90	2.61	2.60	3.36
*R*_F_ (%)	1.71	1.55	1.49	2.39
*χ* ^2^ (%)	1.71	1.54	1.53	2.79

The structural parameters were refined by the standard Rietveld refinement method using the FullProf program.^32^ We utilize the pseudo-Voigt function in order to fit parameters to the experimental data set. The parameters used are: a scale factor, a zero shifting factor, three cell parameters, five shapes and width of the peak factors, one global thermal factor and two asymmetric factors, the background was refined by a linear interpolation between a set background points with refinable heights. The weighted profile factor *R*_wp_, the goodness of fit *χ*^2^, and the difference between the calculated and observed profiles were evaluated at each refinement cycle to determine the refinement quality. The final refinement analysis shows that the experimental spectra and the calculated values obtained by the Rietveld refinement are in good agreement with each other, and all observed peaks have been suitably indexed. The calculated results are shown in Fig. 1(b) and Table 1. One can see from Table 1 that both the lattice parameters (*a* = *b*, and *c*) and unit cell volume show a monotonous decrease with increasing Cu content. Similar structural variation with Cu doping at Mn sites was reported in ref. 24, 26, 29 and 30. (XPS) studies of Cu-doped manganites have shown that Cu ions exist in mixed-valence states: Cu^2+^ and Cu^3+^ with dominant Cu^2+^.^30,31^ The crystal structure and lattice parameters were affected because of the mismatch of ionic radius between the dopant and Mn ions. The B-site ionic radius of Cu^2+^ (0.73 Å) is larger than Mn^3+^ (Mn^4+^) and the ionic radius of Cu^3+^ (0.54 Å) is close to that of Mn^4+^ (0.53 Å) and smaller than the radius of the high spin state of Mn^3+^ (0.645 Å).^33^ Further, it is expected that Cu^2+^ ions substitute Mn^3+^ ions and Cu^3+^ ions substitute Mn^4+^ ions. Furthermore, substitution of Cu^2+^ for Mn^3+^ and Cu^3+^ for Mn^4+^ would lead to a proportionate conversion of Mn^3+^ to Mn^4+^ and Mn^4+^ to Mn^3+^. But the ionic state of Cu^2+^ is expected to be dominant in the samples, therefore, the overall copper doping effect leads to a change in the relative fraction of different valence Mn ions, which results an increase in the number of Mn^4+^ ions in order to preserve charge neutrality and therefore the unit cell volume is found to decrease.

**Fig. 2:**
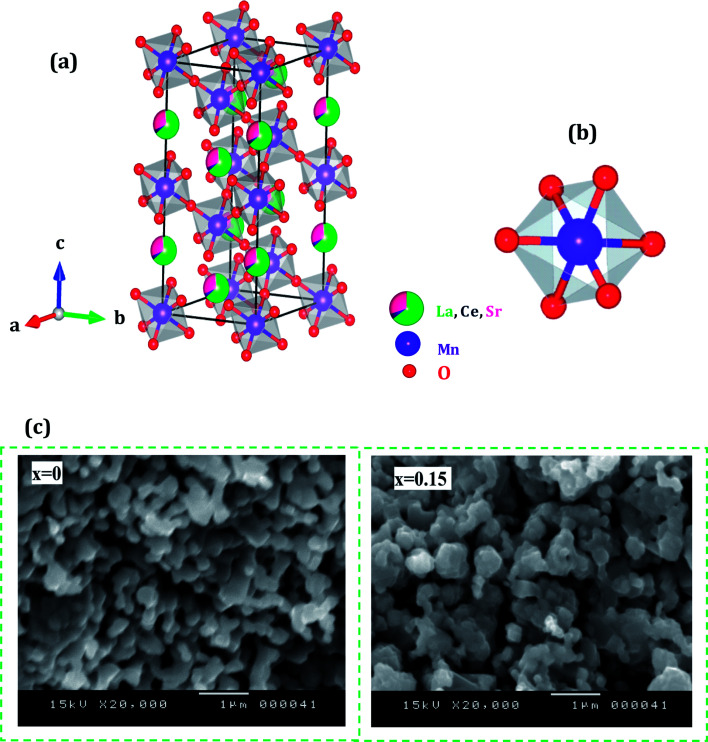
(a) Crystal structure of La_0.65_Ce_0.05_Sr_0.3_MnO_3_ (b) 3D view showing MnO_6_ octahedron (c) SEM images of (*x* = 0 and *x* = 0.15) samples.

Fig. 2(a and b) shows crystal structure of La_0.65_Ce_0.05_Sr_0.3_Mn_1−*x*_Cu_*x*_O_3_ showing MnO_6_ octahedron generated with the help of program VESTA (Visualization for Electronic and Structural Analysis) using refined cell parameters, space group and positional parameters of atoms.^34^ It is represented from the figure that (La/Ce/Sr) cations occupies A-site in ABO_3_ type perovskite structure and are surrounded by 12 oxygen ions while Mn ion occupies the octahedral position (B site) surrounded by six oxygen ions, thus, forming a MnO_6_ octahedron Fig. 2(a).

The surface morphology of samples examined by scanning electron microscopy (SEM) is illustrated in Fig. 2(c). The SEM images show that the particles have an almost homogeneous distribution. The average crystallite size of the samples are obtained by applying the following Rietveld refinement formula 
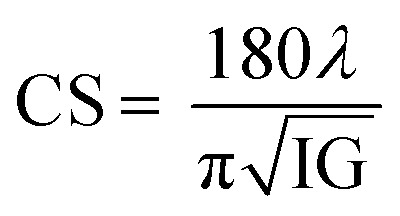
, where *k* is the X-ray wavelength and IG is the Gaussian size parameter given by Rietveld refinement. In all samples a nanometric size for the crystallites is found, between 77 nm and 94 nm (±2 nm), which is related to the moderate synthesis temperatures of these samples, obtained from very reactive precursors from sol–gel procedures. These values are close to those shown by SEM micrographs (the average particles size is ∼100 (±10 nm).

### 3.2. Raman spectroscopy

Raman spectroscopy is a powerful and sensitive tool for the non-destructive investigation and characterization of all kinds of materials. This technique is useful in understanding crystal symmetry, the local structural distortion and its dependence on doping. Our manganites samples shows rhombohedral crystal symmetry using the *R*3̄*c* space group that assumes six equal distances of the Mn–O bonds of MnO_6_ octahedra (Fig. 2(b)). This structure can be described with respect to the ideal cubic structure by considering a rotation of MnO_6_ octahedra about the [111] pseudo cubic diagonal. According to the group theory, for *R*3̄*c* (D_3d_^6^) rhombohedral structure, thirty vibrational degrees of freedom at the *Γ* point are distributed among the irreducible representation as:*Γ* (D_3d_^6^) = 2A_1u_ + 3A_2g_ + A_1g_ + 4A_2u_ + 4E_g_ + 6E_u_

The rhombohedral distortion gives rise to five Raman active modes.

**Fig. 3:**
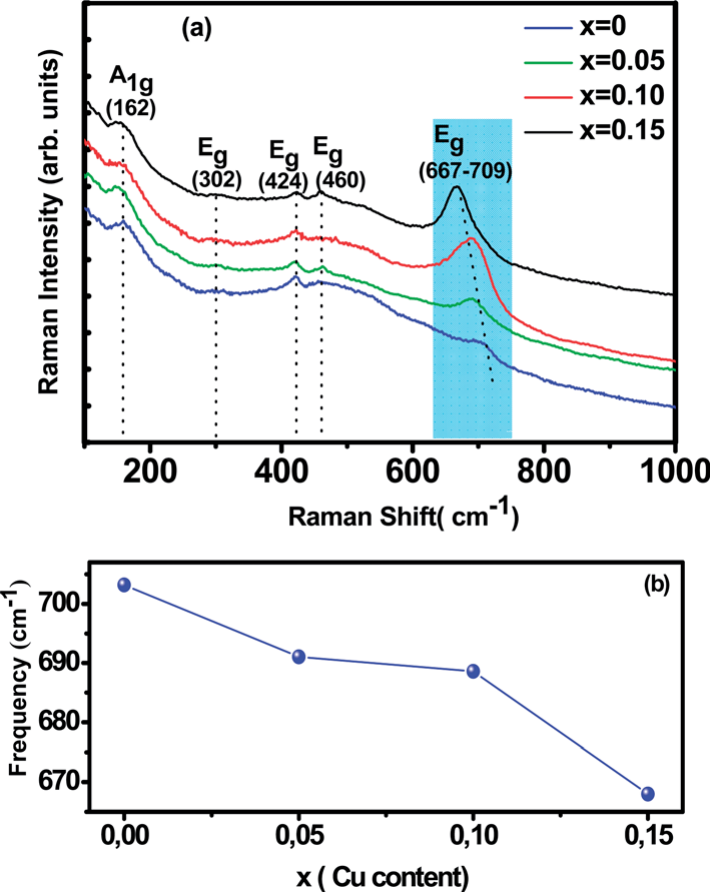
Raman spectrum of La_0.65_Ce_0.05_Sr_0.3_Mn_1−*x*_Cu_*x*_O_3_ (*x* = 0, 0.05, 0.10 and 0.15).

Room temperature Raman spectrum of as synthesized La_0.65_Ce_0.05_Sr_0.3_Mn_1−*x*_Cu_*x*_O_3_ (*x* = 0, 0.05, 0.10 and 0.15) samples in the frequency range of 200–900 cm^−1^ is shown in Fig. 3(a). Five vibration modes have been identified, one (A_1g_) and four (E_g_). These broad bands are located at 162 (A_1g_), 302 (E_g_), 424 (E_g_), 460 (E_g_) and 667–703 (E_g_) cm^−1^, which are associated with rotational-, bending-, and stretching-like vibrations of the MnO_6_ octahedra, respectively.^35,36^ It has been noticed from the graph that with increasing Cu concentration, the Raman scattering intensity of the phonon modes are increasing. The frequencies of the experimental peaks are plotted with doping level for the high-frequency mode E_g_ in Fig. 3(b). This mode shows a substantial shift toward lower frequencies (a downshift of about 30 cm^−1^) as a function of Cu concentration. These shifts are related to the change in the average (Mn/Cu)–O distance.^37^ Similar observations have been reported for some other perovskite compounds with distorted rhombohedral lattice.^35,38,39^

In this work, we underline the E_g_ mode allowed for the symmetric stretching vibration of oxygen in MnO_6_ octahedra. It is reasonable to relate the changes on the E_g_ mode frequency to the modifications of the oxygen octahedral MnO_6_. Moreover, the introduction of substitutional defect in the B-site (like Cu) has a strong effect in the structural changes of the lattice. Since Cu substitution induces a strong local stress, it can be expected that (Mn/Cu)O_6_ octahedra rotate, and the Mn–O bond lengths decrease under this compression.^40^

### 3.3. Magnetic properties

**Fig. 4:**
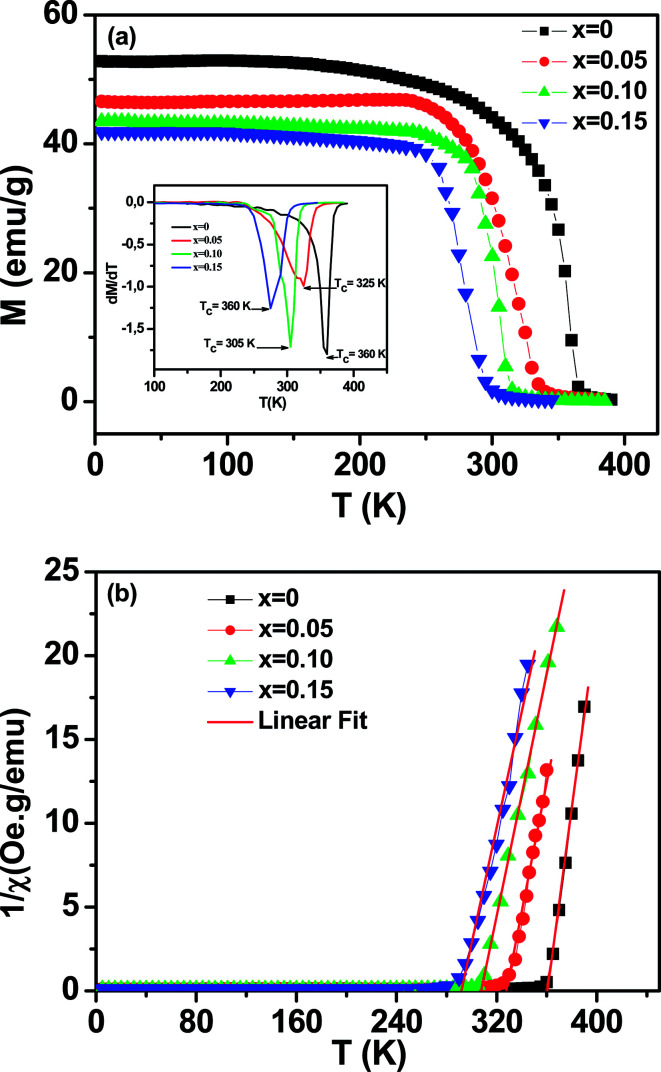
Temperature dependence of the magnetization for La_0.65_Ce_0.05_Sr_0.3_Mn_1−*x*_Cu_*x*_O_3_ (*x* = 0.10 and *x* = 0.15) measured in field cooling (FC) mode at an applied magnetic field of *μ*_0_*H* = 500 Oe. Inset (a) the temperature derivative d*M*/d*T*. (b) Temperature dependence of the inverse of magnetic susceptibility 1 = *χ*. The red line presents the linear fit at high temperature.

The magnetization of La_0.65_Ce_0.05_Sr_0.3_Mn_1−*x*_Cu_*x*_O_3_ (0 ≤ *x* ≤ 0.15) as a function of temperature from 5 K to 400 K under an applied field of 100 Oe is shown in Fig. 4(a).

All samples exhibit a clear transition from paramagnetic to ferromagnetic state with decreasing temperature. The Curie temperature *T*_C_ is the temperature at which the absolute value of d*M*/d*T* is maximum (inset Fig. 4(a)), are summarized in Table 2.

**Table 2:** Values of the Curie temperature *T*_*C*_, the Curie constant *C*, the Curie–Weiss temperature *θ*_P_ and the experimental and theoretical effective paramagnetic moment (*μ*^exp^_eff_) and (*μ*^th^_eff_) for La_0.65_Ce_0.05_Sr_0.3_Mn_1−*x*_Cu_*x*_O_3_ (0 ≤ *x* ≤ 0.15)

	*T*_C_ (K)	*θ*_P_ (K)	*C*	*μ*_eff_ (exp) (μ_B_)	*μ*_eff_ (cal) (μ_B_)^a^	*μ*_eff_ (cal) (μ_B_)^b^
*x* = 0	360	353	2.777	4.71	4.61	4.61
*x* = 0.05	330	323	2.607	4.56	4.45	4.52
*x* = 0.10	305	300	2.769	4.70	4.27	4.43
*x* = 0.15	275	270	2.894	4.81	4.09	4.34

a Under the assumption that all of the Cu ions exist in Cu^2+^ state.

b Under the assumption that all of the Cu ions exist in Cu^3+^ state.

One can see that *T*_C_ decreases monotonically with increasing Cu-doping content. This behavior is consistent with those reported previously on Mn-site substitution of copper.^22,26^ However, the Mn site substitution of Cu^2+^/Cu^3+^ with localized electrons (Cu^2+^: t_2g_^6^ eg^3^, Cu^3+^: t_2g_^6^ eg^2^) reduces the amount of Mn^3+^ (t_2g↑_^3^eg_↑_^1^) with itinerate eg_↑_^1^ electron and converts Mn^3+^ (t_2g↑_^3^e_g↑_^1^) to Mn^4+^ (t_2g↑_^3^_eg↑_^0^) for charge neutrality, directly affecting the DE interaction, weakening the ferromagnetic coupling between Mn^3+^ and Mn^4+^ ions and leads to the lower ordering of FM transition temperature and suppresses the FM interaction. On the other hand, the decrease in *T*_C_ is also closely related to the decrease in the one-electron band-width *W* of e_g_ electron^35^ (Table 1) due to the increase of *d*_Mn/Cu–O_ bond length and the decrease of *θ*_<Mn/Cu–O–Mn/Cu>_ bond angle caused by copper doping. The reduction of the parameter *W* is related to the weakening of the overlap between the O-2p and the Mn-3d orbital, which in turn decreases the (DE) interaction.

To get a clear knowledge about the magnetic interaction for the La_0.65_Ce_0.05_Sr_0.3_Mn_1−*x*_Cu_*x*_O_3_, the magnetic susceptibility (*χ*) could be fitted to the Curie–Weiss law: *χ* = *C*/(*T* − *θ*_p_); where *θ*_p_ is the Curie–Weiss temperature (the temperature at which *χ*^−1^ intercepts the temperature axis) and *C* is the Curie constant were determined by linear fitting of the temperature dependent *χ*^−1^ data in the high temperature paramagnetic region, as displayed in Fig. 4(b) and the values are given in Table 2.

The positive sign of *θ*_p_ values implies the ferromagnetic nature of the magnetic interactions between spins and are basically consistent with the values of *T*_C_.

From the estimated Curie constant (*C*), we have deduced the experimental effective moment (*μ*^exp^_eff_) using the following relation: 
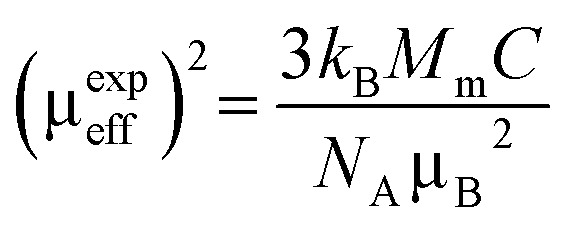
; where *N*_A_ = 6.023 × 10^23^ mol^−1^ is the number of Avogadro, *μ*_B_ = 9.274 × 10^−21^ emu is the Bohr magneton, *M*_m_ is the molecular weight and *k*_B_ = 1.38016 × 10^−16^ erg K^−1^ is the Boltzmann constant.

The theoretical effective paramagnetic moment (*μ*^th^_eff_) was calculated using the calculated Mn^3+^/Mn^4+^ contents under the assumption that all the Cu ions exist in either Cu^2+^ or Cu^3+^ state. The spin-only magnetic moments for free Mn^3+^, Mn^4+^, Cu^2+^ and Cu^3+^ are 4.89*μ*_B_, 3.87*μ*_B_, 1.73*μ*_B_, 2.83*μ*_B_, respectively. The obtained values of (*μ*^exp^_eff_) and (*μ*^th^_eff_) are listed in Table 2. The experimental (*μ*^exp^_eff_) value is little larger than the calculated value using the spin-only moment. Such a difference in (*μ*_eff_) value may be ascribed to the appearance of short-range FM interactions in the paramagnetic state. This result is commonly observed in manganites.^8,41,42^

**Fig. 5:**
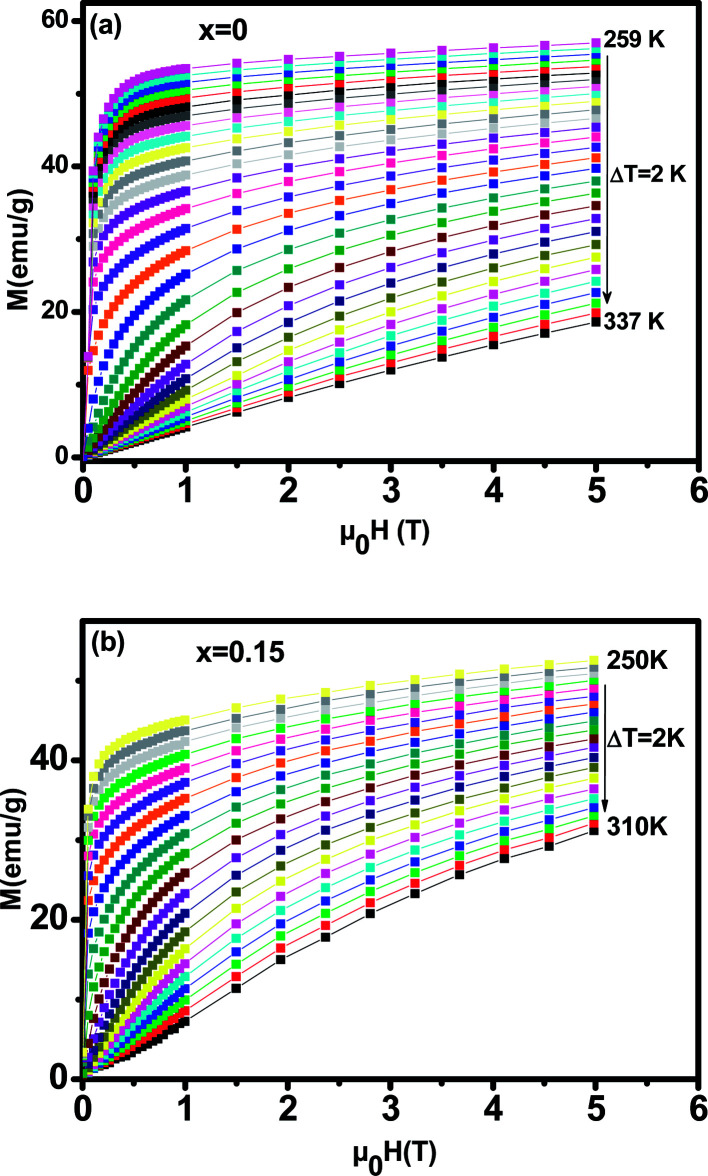
Isothermal magnetization *versus* magnetic field around *T*_C_ of La_0.65_Ce_0.05_Sr_0.3_Mn_1−*x*_Cu_*x*_O_3_, (a) for *x* = 0 and (b) for *x* = 0.15.

In order to investigate the magnetic behavior at low temperatures, we have performed magnetization measurements as a function of the applied magnetic field *μ*_0_*H* up to 5 T at various temperatures. We plot in Fig. 5 the magnetization evolution *versus* the applied magnetic field obtained at different temperature (isothermal magnetization) for (a) *x* = 0, and (b) *x* = 0.15 samples. At a given lower fields, (*M*–*H*–*T*) curves show a rapid increase and get saturated at higher fields. For all the studied samples, the magnetization has been found to increase with decreasing temperature in the selected temperature range, where thermal fluctuation of spins decreases with decreasing temperature.

**Fig. 6:**
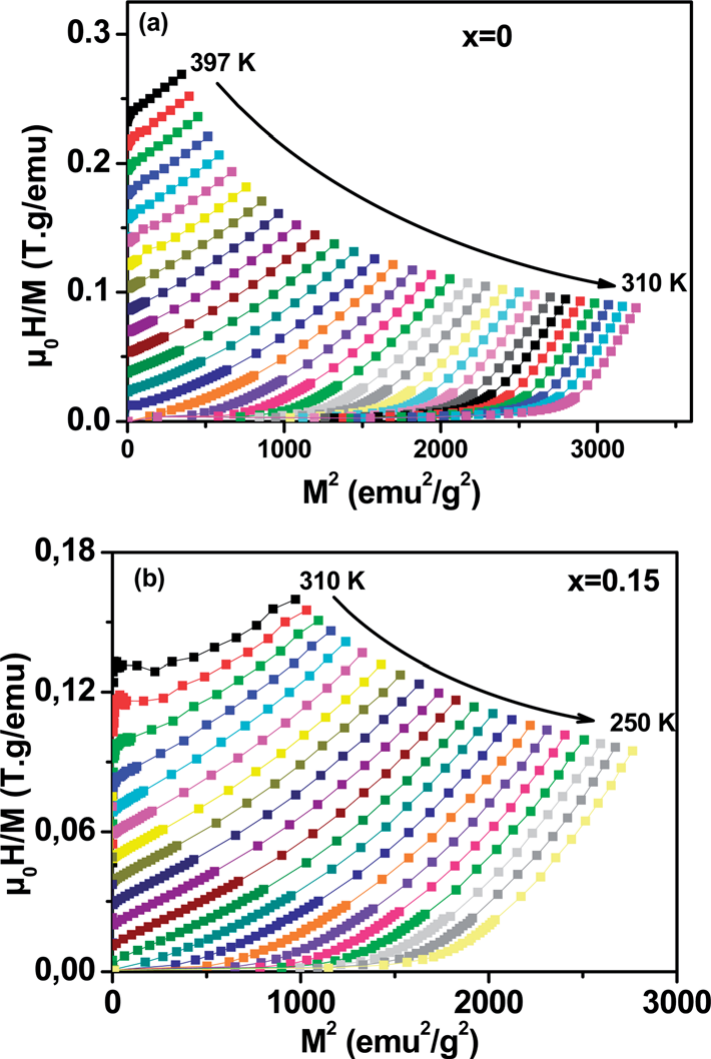
Arrott plot of *μ*_0_*H*/*M vs. M*^2^ at different temperatures for La_0.65_Ce_0.05_Sr_0.3_Mn_1−*x*_Cu_*x*_O_3_, (a) *x* = 0 and (b) *x* = 0.15.

To determine the nature of the FM–PM phase transition (first or second order) for our samples, we plotted in Fig. 6(a and b) the Arrott plot^43^ (*μ*_0_*H*/*M versus M*^2^) for *x* = 0 and *x* = 0.15. According to the Banerjee criterion,^44^ all of the *M*^2^*vs. μ*_0_*H*/*M* curves show positive slopes without infection points, which is characteristic of second order transitions.

### 3.4. Magnetocaloric effect and second-order magnetic phase transition

MCE is an intrinsic property of magnetic materials. It is the response of the material toward the application or removal of a magnetic field. This response is maximized when the material is near its magnetic ordering temperature. In an isothermal process, the magnetic entropy change of the materials can be derived from the Maxwell relation as shown below:^45^2
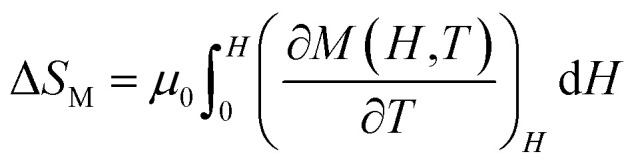


**Fig. 7:**
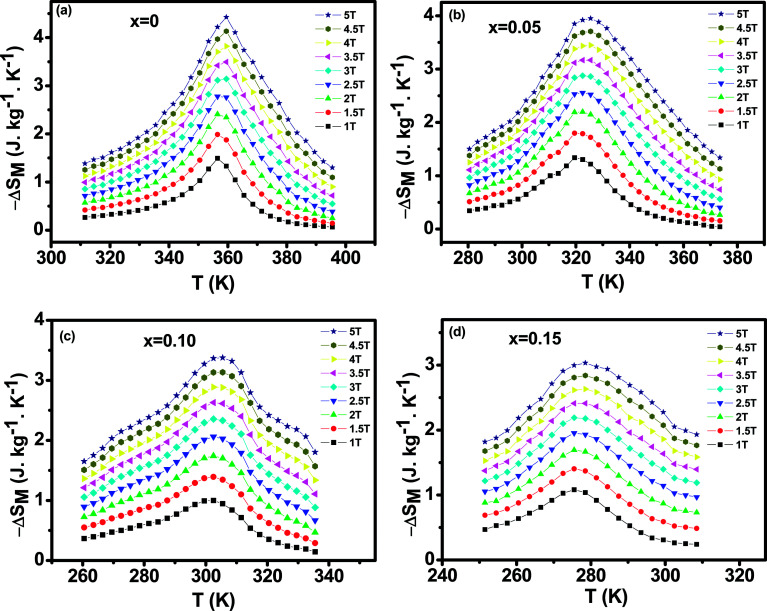
The temperature dependence of the magnetic entropy change (Δ*S*_M_) under different applied magnetic fields (a) for *x* = 0, (b) for *x* = 0.05, (c) for *x* = 0.10 and (d) for *x* = 0.15.

The magnetic entropy changes, Δ*S*_M_, of La_0.65_Ce_0.05_Sr_0.3_Mn_1−*x*_Cu_*x*_O_3_ (*x* = 0, 0.05, 0.1 and 0.15) have been calculated using the Maxwell relation^46^ and are plotted in Fig. 7 as a function of temperature and field.

The maximum value of magnetic entropy change Δ*S*_M_ is found to be around *T*_C_ and it increases with increasing the magnetic applied field due to the enhancement of FM interactions. As the Cu content increases the magnitude of Δ*S*_M_ decreases under a given field strength. Indeed, under the magnetic field change from 0 to 5 T, the values of |Δ*S*^max^_M_| observed for *x* = 0.00, 0.05, 0.1 and *x* = 0.15 are found to be 4.43, 5.15, 3.37 and 3.03 J kg^−1^ K^−1^, respectively. The value of |Δ*S*^max^_M_| for *μ*_0_*H* = 1 and 5 T are listed in Table 2 along with related compounds^47,48^ for easy comparison.

Relative cooling power (RCP) is another important parameter to quantify the efficiency of the magnetocaloric material. It is a measure of how much heat can be transferred between the cold and the hot tanks in one ideal refrigeration cycle. It can be defined as

3 RCP = −Δ*S*^max^_M_ × *δ*_FWHM_,

where *δ*_FWHM_*=* Δ*T* is the full-width at half maximum peak and −Δ*S*^max^_M_ is the maximum value of magnetic entropy change which is occurred at Curie temperature.

The RCP values of the La_0.65_Ce_0.05_Sr_0.3_Mn_1−*x*_Cu_*x*_O_3_ are evaluated under the magnetic field changes of 1 and 5 T. For a comparison *T*_C_ and magnetocaloric parameters of some relevant manganites in the literature are listed in Table 3.

**Table 3:** Maximum entropy change |Δ*S*^max^_M_| and relative cooling power (RCP), for La_0.65_Ce_0.05_Sr_0.3_Mn_1−*x*_Cu_*x*_O_3_ (0 ≤ *x* ≤ 0.15), occurring at the Curie temperature (*T*_C_) and under magnetic field variations, *μ*_0_*H* = 1 T or *μ*_0_*H* = 5 T, compared to several materials considered for magnetic refrigeration

Composition	*T*_C_ (K)	Δ*H* (T)	|−Δ*S*^max^_M_ (J kg^−1^ K^−1^)|	RCP (J kg^−1^)	Ref.
Gd	293	1	3.25	—	47
La_0.77_Sr_0.23_Mn_0.9_Cu_0.1_O_3_	325	1	4.41	57	26
La_0.7_Sr_0.3_Mn_0.9_Cu_0.1_O_3_	347	1	3.24	—	21
La_0.65_Ce_0.05_Sr_0.3_MnO_3_	360	1	1.49	33	This work
La_0.65_Ce_0.05_Sr_0.3_Mn_0.95_Cu_0.05_O_3_	330	1	1.34	44	This work
La_0.65_Ce_0.05_Sr_0.3_Mn_0.9_Cu_0.1_O_3_	305	1	1.5	36	This work
La_0.65_Ce_0.05_Sr_0.3_Mn_0.85_Cu_0.15_O_3_	275	1	1.08	28	This work
La_0.67_Ba_0.33_Mn_0.98_Ti_0.02_O_3_	310	1	0.93	45	17
Gd	293	5	9.5	410	48
La_0.65_Ce_0.05_Sr_0.3_MnO_3_	360	5	4.43	132	This work
La_0.65_Ce_0.05_Sr_0.3_Mn_0.95_Cu_0.05_O_3_	330	5	3.95	175	This work
La_0.65_Ce_0.05_Sr_0.3_Mn_0.9_Cu_0.1_O_3_	305	5	3.37	102	This work
La_0.65_Ce_0.05_Sr_0.3_Mn_0.85_Cu_0.15_O_3_	275	5	3.06	88	This work
La_0.65_Eu_0.05_Sr_0.3_Mn_0.9_Cr_0.1_O_3_	310	5	3.35	207	16
La_0.67_Ba_0.33_Mn_0.98_Ti_0.02_O_3_	310	5	3.19	307	17

The maximum value of RCP is obtained for *x* = 0.05, which is 43% of that of pure Gd, the prototype magnetic refrigerant material (RCP = 410 J kg^−1^).^48^ Our results indicate that this compound is promising for room temperature magnetic refrigeration.

For further clarification of the phase transition of the samples as an alternative to the Banerjee criterion^44^ we used the phenomenological universal curve method, proposed by Franco *et al.*,^49,50^ which is a general approach to determine the order of the phase transition. In order to construct this phenomenological universal curve, an analogous procedure to that described in ref. 51 has been used. It consists in normalizing the Δ*S*_M_ curves with respect to their maximum and rescaling the temperature axis as4
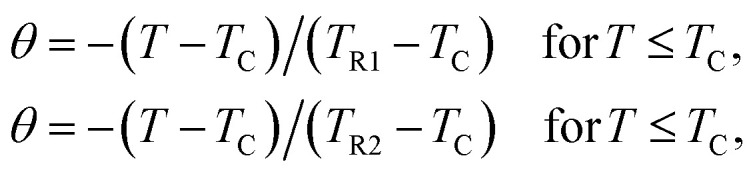
where *T*_R1_ and *T*_R2_ are the two reference temperatures corresponding to the half maximum of Δ*S*_M_(*T*_R1_) = Δ*S*_M_(*T*_R2_) = Δ*S*^max^_M_/2. For the materials undergoing second order magnetic phase transition, the rescaled magnetic entropy change curves should follow a universal behavior. While the scaled Δ*S*_M_ curves do not collapse as a single curve, the materials undergo a first-order phase transition.^42^

**Fig. 8:**
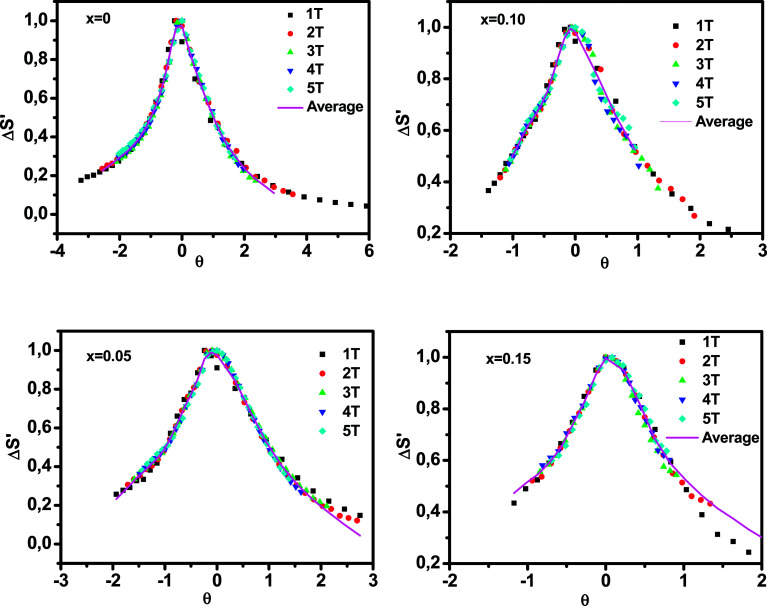
Normalized Δ*S*_M_*versus* rescaled temperature *θ* for La_0.65_Ce_0.05_Sr_0.3_Mn_1−*x*_Cu_*x*_O_3_, the solid line is the average curve.

Fig. 8 shows the universal curves constructed for the experimental Δ*S*_M_ (*T*, *H*) curves as a function of the rescaled temperature for La_0.65_Ce_0.05_Sr_0.3_Mn_1−*x*_Cu_*x*_O_3_ (*x* = 0, 0.10 and 0.15) samples. It can be clearly seen from this figure that all normalized entropy change curves collapse into a single universal curve, which confirms that the PM–FM phase transition observed for our compounds is of a second-order. Hence, this result is consistent with the trends observed in the Arrott plots (Fig. 6).

The solid line corresponds to the average of the universal scaling. This average curve, once the temperature axis is back transformed from the reduced temperature to the unnormalized one, allows making extrapolations to lower temperatures for the high field data and obtaining a finer description of the peak for the low field curves.^52,53^

## 4. Conclusion

In summary, we have studied the effect of copper doping lanthanum manganite ions on structural, magnetic and magnetocaloric properties of La_0.65_Ce_0.05_Sr_0.3_Mn_1−*x*_Cu_*x*_O_3_ (0 ≤ *x* ≤ 0.15) prepared using the Pechini sol–gel method. Rietveld refinement of XRD patterns shows that all samples crystallized in a rhombohedral structure with *R*3̄*c* space group. The Cu-doping induces the suppression of the one-electron band-width *W* of e_g_ electron due the variations of the bond length and bond angle, leading to destruction of the DE interaction. The Curie temperature and the maximum magnetic entropy change decrease with the increase in the Cu content. This is attributed to the structural distortion of MnO_6_ octahedron and the changes in the valence states of the Cu and Mn ions upon Cu doping, weakening the ferromagnetic exchange interaction. A uniform phenomenological function that describes the magnetic entropy change is found for these materials, which provides good handle on designing of magnetocaloric materials for micro magnetic refrigerators.

The La_0.65_Ce_0.05_Sr_0.3_Mn_0.95_Cu_0.05_O_3_ sample is found to have a comparable MCE around 330 K with a maximum Δ*S*_M_ of 1.34 J kg^−1^ K^−1^ and a RCP of 44 J kg^−1^ under a magnetic field change of 1 T, and can be considered as competitive candidate for magnetic refrigerant materials operating near room temperature.

## Conflicts of interest

There are no conflicts to declare.

## Supplementary Material
